# Overcoming the drug resistance barrier: progress in fosfomycin combination therapy against multidrug-resistant pathogens

**DOI:** 10.3389/fmicb.2025.1702881

**Published:** 2025-11-18

**Authors:** Yan Wu, Jimin Li, Fengling Qiao, Jinlin Guo, Lin Zhang, Xu Jia

**Affiliations:** 1 College of Medical Technology, Chengdu University of Traditional Chinese Medicine, Chengdu, China; 2 Chongqing Key Laboratory of Sichuan-Chongqing Co-construction for Diagnosis and Treatment of Infectious Diseases Integrated Traditional Chinese and Western Medicine, Chengdu, China; 3 Key Laboratory of Non-coding RNA and Drug Discovery at Chengdu Medical College, Chengdu, China; 4 Department of Pharmacy, Shaoxing People’s Hospital, Shaoxing, Zhejiang, China; 5 School of Basic Medical Sciences, Chengdu Medical College, Chengdu, China

**Keywords:** fosfomycin, combination therapy, antimicrobial resistance, multidrug resistance, antimicrobial co-administration

## Abstract

In the intensifying global crisis of antimicrobial resistance (AMR), the “old” antibiotic fosfomycin has regained prominence because of its unique mechanism of action and potent activity against numerous multidrug-resistant (MDR) pathogens. However, its clinical application is hampered by the rapid emergence of resistance during monotherapy. Rational combination therapy represents a strategic necessity to preserve and enhance the efficacy of fosfomycin. This review systematically analyzes the antibacterial and molecular mechanisms of resistance to fosfomycin, with a focus on the growing threat posed by plasmid-mediated resistance genes. The preclinical and clinical evidence of key combination regimens (including β-lactams, aminoglycosides, fluoroquinolones, polymyxins, and daptomycin) has been comprehensively evaluated, with detailed discussions of the mechanistic foundations for the observed synergistic effects. Although *in vitro* and animal models show substantial promise, we critically examine the translational gap between positive preclinical results and clinical realities, discussing major barriers to clinical advancement. Finally, we outline a prospective research agenda, encompassing pharmacokinetic/pharmacodynamic (PK/PD)-guided precision dosing, exploring non-antibiotic adjuvants, and developing more predictive preclinical models to unlock the full potential of fosfomycin-based combinations against MDR infections.

## 1 Introduction

Antimicrobial resistance (AMR) has emerged as a significant global public health threat, as highlighted by organizations such as the US Centers for Disease Control and Prevention (CDC) and the World Health Organization (WHO) (Castanheira et al., 2023). The relentless emergence of multidrug-resistant (MDR), extensively drug-resistant (XDR), and even pandrug-resistant (PDR) pathogens severely limits clinical therapeutic options, necessitating urgent innovative strategies (Falagas et al., 2016). According to CDC data, at least 2.8 million antibiotic-resistant infections occur in the United States each year, resulting in more than 35,000 deaths (Morrison and Zembower, 2020). This crisis has prompted scientific and medical communities to reevaluate the existing antimicrobial arsenal.

Faced with the depletion of novel antibiotic development channels, the repurposing and optimized use of previously neglected “old drugs” has become a key strategy (Dijkmans et al., 2017). The use of fosfomycin, a natural antibiotic discovered more than 45 years ago, has experienced a significant revival because of its unique mechanism of action and potent activity against numerous MDR pathogens. Its broad-spectrum bactericidal activity and extremely favorable toxicity profile make it an attractive option for addressing complex infections (Dijkmans et al., 2017; Silver, 2017). However, the application of fosfomycin faces a central paradox: despite being a potent bactericide with good safety, the rapid development of resistance during both *in vitro* and *in vivo* monotherapy severely restricts its clinical utility (Silver, 2017). This contradiction highlights the need to transition from monotherapy to rational combination therapies. Indeed, the renewed focus on fosfomycin presents a double-edged sword. Its activity against MDR bacteria also creates selective pressure for the emergence and spread of fosfomycin resistance. With the increasing use of fosfomycin, particularly in the treatment of multidrug-resistant infections, its resistance rate is also rising, a trend confirmed by usage data from regions such as Spain (Gardiner et al., 2019), which establishes a concerning feedback loop: the AMR crisis drives fosfomycin use, which in turn contributes to the emergence of the novel problem of fosfomycin resistance. This dynamic suggests that the only viable approach to breaking this cycle lies in strategies that suppress resistance development, with combination regimens being central to this effort. Research indicates that combination therapy is not only a multi-drug option but also a strategic necessity to enhance bactericidal activity through co-administration, suppressing the emergence and selection of resistance, and potentially restoring susceptibility in strains resistant to antibacterial drugs (Antonello et al., 2020).

This review aims to provide basic microbiology researchers with a detailed report, systematically dissecting the mechanistic basis, preclinical evidence, and clinical results of fosfomycin combination therapy while also exploring future research directions.

## 2 Pharmacology of fosfomycin

### 2.1 Unique chemical structures and formulations

Fosfomycin [chemical name: (1R,2S)-epoxypropylphosphonic acid] is an extremely small (138 g/mol), highly polar molecule that was first isolated from Streptomyces cultures in 1969 and is characterized by high tissue penetration and low toxicity (Hendlin et al., 1969; Popovic et al., 2010). Its structural similarity to phosphoenolpyruvate (PEP) underlies its broad-spectrum antibacterial activity against both gram-positive and gram-negative bacteria. Clinically, two major salt formulations of FOS (C_3_H_7_O_4_P) are utilized: oral fosfomycin trometamol (C_3_H_7_O_4_P ⋅ C_4_H_11_NO_3_), with a bioavailability of 34%–58%, and intravenous fosfomycin disodium (C_3_H_5_Na_2_O_4_P), which is employed to achieve higher systemic concentrations (Kwan and Beahm, 2020; Marino et al., 2022). Trometamol, an alkaline organic compound, is believed to mitigate acid-catalyzed hydrolysis. Compared with the oral calcium salt formulation (bioavailability 12%–37%), fosfomycin trometamol achieves serum concentrations 2- to 4-fold greater, establishing it as the preferred oral formulation for treating uncomplicated urinary tract infections (UTIs) (Bergan, 1990; Dijkmans et al., 2017; Neuner et al., 2012). Fosfomycin disodium is currently approved in many countries for the treatment of soft tissue infections and sepsis (Michalopoulos et al., 2011).

### 2.2 Direct bactericidal action

**FIGURE 1:**
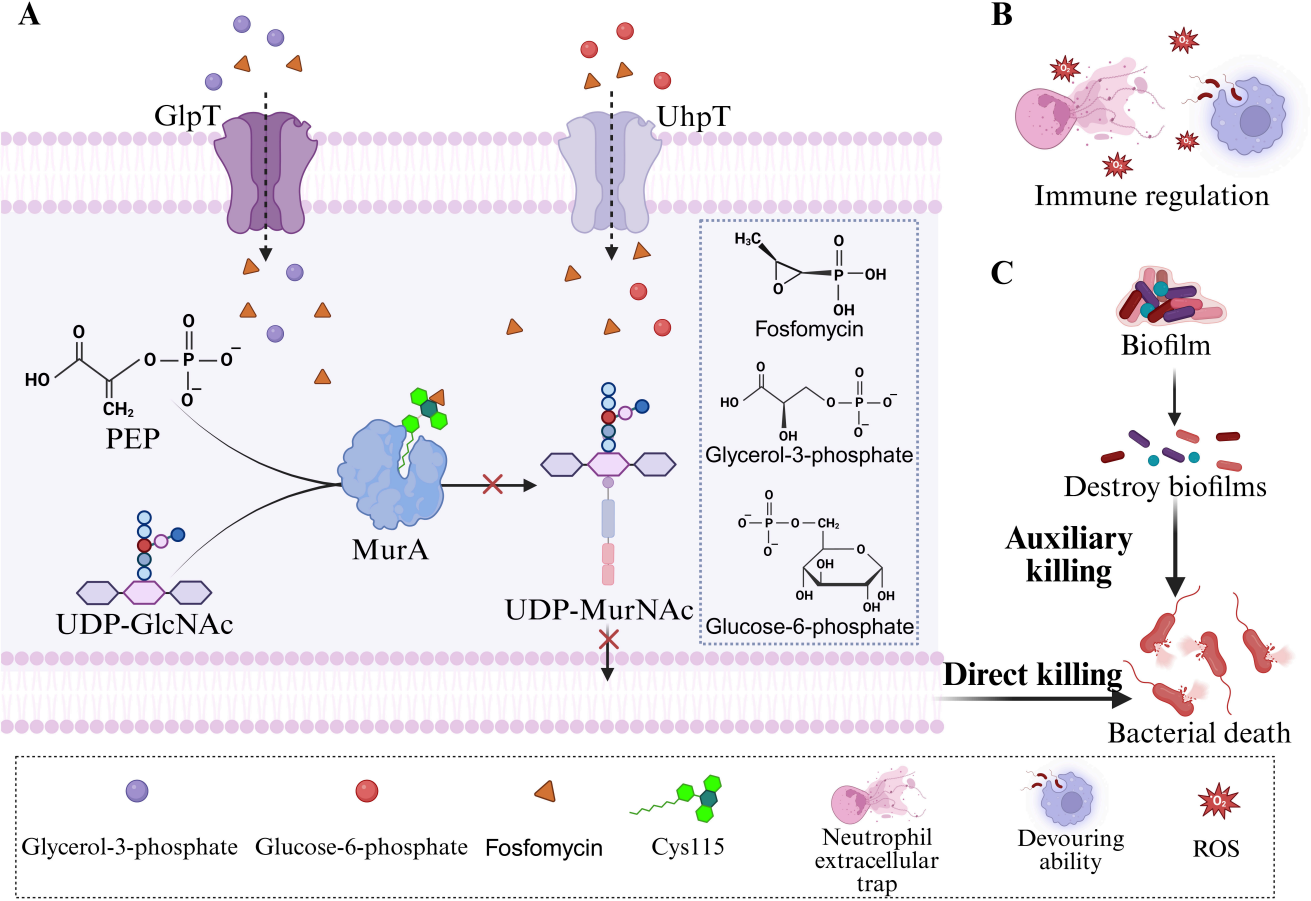
Antibacterial mechanism of fosfomycin. **(A)** FOS inhibits the formation of UDP-MurNAc, thereby exerting a direct bactericidal effect on bacteria. **(B)** FOS exerts an indirect bactericidal effect on bacteria through immunomodulation. **(C)** FOS exerts an indirect bactericidal effect by destroying bacterial biofilms.

Unlike other mainstream antibiotics, fosfomycin (FOS) exerts its core mechanism by interfering with the first step of bacterial cell wall synthesis: the formation of the peptidoglycan precursor UDP-N-acetylmuramic acid (UDP-MurNAc), which acts earlier than β-lactams and glycopeptides (Figure 1A; Borisova et al., 2014; Falagas et al., 2016). FOS acts on the bacterial cytoplasm (Kahan et al., 1974). Once inside the cytoplasm, FOS functions as a structural analog of phosphoenolpyruvate (PEP) and covalently binds to the active site Cys115 of MurA (UDP-GlcNAc enolpyruvyl transferase), thereby inactivating the MurA enzyme (Aghamali et al., 2019). MurA catalyzes the condensation of UDP-N-acetylglucosamine (UDP-GlcNAc) with PEP to form UDP-MurNAc (Silver, 2017). Once the MurA enzyme is inhibited, UDP-MurNAc cannot be produced, ultimately leading to bacterial cell lysis. The interaction between FOS and MurA is highly specific, contributing to the low toxicity of this complex in mammals.

To enter cells, FOS relies primarily on two transport systems in *Escherichia coli*: GlpT (glycerol-3-phosphate transporter) and UhpT (hexose-6-phosphate transporter) (Castañeda-García et al., 2013). The expression of these transporters is induced by their respective substrates, glycerol-3-phosphate (G3P) and glucose-6-phosphate (G6P). Extracellular G3P and G6P enter bacterial cells via GlpT and UhpT, respectively, and induce high expression of GlpT and UhpT in the presence of the cAMP-CRP complex (Castañeda-García et al., 2013; Yang et al., 2016). This characteristic explains why the addition of G6P to the culture medium is essential for reliable fosfomycin susceptibility testing. G6P supplementation enhances the expression of the UhpT system, ensuring adequate drug uptake and generating accurate minimum inhibitory concentration (MIC) values.

### 2.3 Auxiliary bactericidal effects

#### 2.3.1 Immunomodulation

In addition to its core bactericidal mechanism, FOS has auxiliary properties that are beneficial for clinical therapy. Notably, FOS exerts complex modulatory effects on the host immune system. Studies indicate that FOS enhances the phagocytic and bactericidal capacity of phagocytes (neutrophils and macrophages) against invading pathogens, including promoting phagocytosis, inducing reactive oxygen species (ROS) generation, and stimulating the production of extracellular traps (ETs) (Figure 1B; Shen et al., 2016). FOS inhibits the production of IL-2 in T cells, leukotriene B4 (LTB4) in neutrophils, and the expression of IL-8 mRNA in monocytes (Honda et al., 1998; Morikawa et al., 1993). Furthermore, FOS has been shown to modulate the production of proinflammatory cytokines, such as tumor necrosis factor (TNF)-α, IL-1β, and IL-6, *in vivo* (Matsumoto et al., 1999). However, a study evaluating the effects of FOS on proinflammatory cytokines in healthy volunteers revealed that the protein and mRNA expression levels of TNF-α, IL-1β, and IL-6 exhibited little difference in the presence of FOS (Sauermann et al., 2007). Although the clinical significance of these effects is still being explored, they suggest that fosfomycin may confer dual benefits by modulating host immune and inflammatory responses.

#### 2.3.2 Anti-biofilm activity

Fosfomycin also effectively penetrates and disrupts biofilms formed by various pathogens, including *Staphylococcus aureus*, *Staphylococcus epidermidis*, *Pseudomonas aeruginosa*, and *Enterococcus* spp. (Figure 1C; Descourouez et al., 2013; Hajdu et al., 2009; Mikuniya et al., 2007; Shi et al., 2014). Multiple *in vitro* studies have shown that FOS, either alone or in combination with other antimicrobial agents, not only reduces or eradicates clinically significant bacteria within biofilms but also induces structural alterations in biofilms (Ai et al., 2025; Anderson et al., 2013; Mihailescu et al., 2014). For example, in a recent *in vitro* biofilm infection model, the combination of fosfomycin and daptomycin exhibited superior antibiofilm activity, demonstrating synergistic antibacterial effects against methicillin-resistant *Staphylococcus aureus* (MRSA) (Ai et al., 2025). In a rat urinary tract infection model, scanning electron microscopy revealed that the combination of fosfomycin and prulifloxacin disrupted and eliminated multilayered *P. aeruginosa* biofilms from polyethylene tube surfaces (Mikuniya et al., 2007). Fosfomycin monotherapy has also been reported to reduce the density of *S. epidermidis* biofilms (Hajdu et al., 2009). Furthermore, combinations of fosfomycin with traditional Chinese medicines have shown significant potential in combating biofilms (Chrisostomo et al., 2025). The combination of fosfomycin and cryptotanshinone was found to inhibit biofilm formation by fosfomycin-resistant *S. aureus*, thereby reducing fosfomycin resistance (Ruan et al., 2020). Collectively, the antibiofilm activity of fosfomycin highlights its considerable potential for treating chronic infections and infections associated with medical devices, providing strong support for its use in combination therapeutic regimens.

## 3 Molecular mechanisms of fosfomycin resistance

A thorough understanding of how bacteria evade the action of fosfomycin is fundamental for designing effective combination therapies to overcome antibiotic resistance. The mechanisms of fosfomycin resistance are broadly categorized into two classes: chromosomally mediated resistance and plasmid-mediated resistance (Table 1; Hosoi et al., 2024). Chromosomally mediated resistance typically arises from mutations in a bacterium’s genes and is transmitted vertically to its progeny. In contrast, plasmid-mediated resistance involves bacteria acquiring mobile genetic elements (such as plasmids) carrying the fos gene through horizontal gene transfer. These genes encode enzymes that modify and inactivate fosfomycin (Mattioni Marchetti et al., 2023a).

**TABLE 1:** Overview of the resistance mechanisms of fosfomycin.

Mechanism category	Specific mechanism	Molecular effect	Associated pathogens	FOS MIC (ug/ml)	References
Impaired fosfomycin uptake	*glpT* and *uhpT* mutations	The decreased activity of GlpT and UhpT	MRSA	128∼>1024	Fu et al., 2015
	*glpT* and *uhpT* deficiency	Loss of GlpT and UhpT	*S. aureus*	>1024	Xu et al., 2017
	*glpT* mutation	The decreased activity of GlpT	*E. coli*	>128	Mattioni Marchetti et al., 2023b; Seok et al., 2020
	*glpT*, *uhpA*, *CyaA* mutations	The decreased activity of GlpT and UhpT	*E. coli*	\	Lucas et al., 2018
	*glpT* and *uhpA* mutations	The decreased activity of GlpT and UhpT	*Klebsiella pneumoniae*	128∼512	Lu et al., 2016
Fosfomycin target modification	*murA* mutation	MurA overexpression, reduced drug concentration	*E. coli*	128	Couce et al., 2012
	*murA* mutation	Lower drug affinity	*E. coli*	\	Takahata et al., 2010; Wu and Venkateswaran, 1974
Production of fosfomycin-modifying enzymes	*fosA*	Glutathione-dependent drug inactivation	*Serratia marcescens*	\	Mendoza et al., 1980
	*fosA* ^KG^	Glutathione-dependent drug inactivation	*E. coli*	>1024	Ito et al., 2018
	*fosA2*	Glutathione-dependent drug inactivation	*Enterobacter cloacae*	\	Xu et al., 2011
	*fosA3*	Glutathione-dependent drug inactivation	*E. coli*	>512	Loras et al., 2020
	*fosA3*	Glutathione-dependent drug inactivation	*E. coli*	>256	Wachino et al., 2010
	*fosA3*, *fosA4*, *fosA6*	Glutathione-dependent drug inactivation	*E. coli*	>256	Sadek et al., 2022
	*fosA5*	Glutathione-dependent drug inactivation	*E. coli*	512	Ma et al., 2015
	*fosA8*	Glutathione-dependent drug inactivation	*E. coli*	\	Poirel et al., 2019
	*fosB*	Bacillithiol/L-cysteine-dependent drug inactivation	MRSA	>128	Fu et al., 2015
	*fosC2*	Catalytic drug phosphorylation	*E. coli*	>256	Wachino et al., 2010
	*fosX*	Drug inactivation catalyzed by epoxide hydrolase	*Acinetobacter baumannii*	32∼≥256	Leite et al., 2021
	*fosL1*	Glutathione-dependent drug inactivation	*E. coli*	>1024	Kieffer et al., 2020

### 3.1 Chromosomally mediated resistance

#### 3.1.1 Impaired fosfomycin uptake

Mutations (such as insertions, deletions, or truncations) in the transporter-encoding genes *glpT* and *uhpT* result in impaired function or complete loss of the GlpT and UhpT transporters, respectively, thereby blocking or reducing fosfomycin uptake (Castañeda-García et al., 2013). The expression of these genes (*glpT*, *uhpT*) requires the presence of cAMP. Mutations can reduce cAMP levels in the *ptsI* or *cyaA* genes, which also affect the catabolism of various carbohydrates. Furthermore, high-level expression of the *uhpT* gene requires the regulatory gene *uhpA* (Falagas et al., 2019). Mutations in any of these regulatory pathway genes diminish antibiotic uptake, leading to varying degrees of fosfomycin resistance (Nilsson et al., 2003). Notably, such mutations often incur a significant “fitness cost,” such as the inability of bacteria to metabolize carbon sources such as G3P and G6P. This may explain why the clinical resistance rate for treating uncomplicated urinary tract infections is considerably lower than that observed *in vitro* (Silver, 2017). However, for some pathogens, such as *P. aeruginosa*, GlpT is the sole functional fosfomycin transporter; consequently, the fitness cost associated with its mutation might be lower, making it a clinically significant resistance mechanism (Pipitone et al., 2023). In *Listeria monocytogenes*, bacteria exhibit intrinsic resistance to fosfomycin due to the absence of antibiotic transporters. However, the virulence factor Hpt (a glucose-6-phosphate permease) mediates fosfomycin uptake, conferring antibiotic susceptibility during infection (Castañeda-García et al., 2013).

#### 3.1.2 Fosfomycin target modification

Modification of the antibiotic target MurA constitutes another mechanism leading to fosfomycin resistance. Mutations in the *murA* gene, which encodes the MurA enzyme, can result in the substitution of the active site cysteine (Cys115) with other amino acids, such as Cys115Asp or Cys115Glu, thereby reducing its affinity for fosfomycin (Kim et al., 1996). Additionally, overexpression of the *murA* gene can dilute the intracellular fosfomycin concentration, effectively lowering the drug level within the cell and enabling bacteria to acquire resistance at a low fitness cost (Couce et al., 2012). Due to the absence of MurA, *Pseudomonas* species are considered to have inherent resistance to fosfomycin; however, reports indicate variability in fosfomycin activity, with 61% of *P. aeruginosa* isolates being susceptible to fosfomycin (MIC ≤ 64 μg/ml) (Humphries et al., 2021). However, mutations in the *murA* gene are rare in clinical isolates. This rarity is attributed to the isolation of murA mutants in mutagenized *E. coli* by Wu and Venkateswaran (1974) and the identification of two murA mutants in a Japanese study of clinical *E. coli* isolates (Takahata et al., 2010).

### 3.2 Plasmid-mediated resistance

Another critical pathway for clinical fosfomycin resistance is the plasmid-mediated production of modifying enzymes. Diverse fosfomycin-modifying enzymes, including FosA and its variants (FosA1–FosA10), FosB, FosC2, FosX, and FosL1–L2, have been identified (Zhang et al., 2025). These enzymes are encoded by the *fos* gene (located on plasmids) and catalyze the opening of the epoxide ring of fosfomycin, rendering it inactive. FosA (glutathione S-transferase) is the most prevalent modifying enzyme and is widespread in the Enterobacteriaceae. It inactivates fosfomycin by catalyzing the addition of glutathione to its epoxide ring. Among these, the FosA3 variant is the most widely disseminated acquired fosfomycin resistance determinant and is speculated to originate from *Kluyvera georgiana* (Ito et al., 2018; Yu et al., 2024). According to a study of 350 strains of extended-spectrum β-lactamase (ESBL)-producing *E. coli* in Mexico, 60.5% of fosfomycin-resistant strains harbored *fos* genes, with 60% of these carrying *fosA3* (Galindo-Méndez et al., 2022). FosB (bacillithiol/L-cysteine transferase), which is primarily found in gram-positive bacteria such as Staphylococci, Bacilli, and Enterococci, inactivates fosfomycin by adding bacillithiol or L-cysteine (Bruce et al., 2022; DiCicco et al., 2014; Haenni et al., 2012). FosC2, an enzyme structurally similar to FosA, catalyzes the phosphorylation of fosfomycin in the presence of ATP to inactivate it (Wachino et al., 2010). FosX (epoxide hydrolase) functions similarly to other Fos enzymes, inactivating fosfomycin by adding water at the C1 position to open the epoxide ring (Castañeda-García et al., 2013). FosL1 and FosL2 are recently described novel glutathione-S-transferases with 63% identity to FosA8, exhibiting high-level resistance to fosfomycin (Kieffer et al., 2020).

The most concerning trend in fosfomycin resistance today is not only the presence of fos genes but also their frequent colocalization on the same plasmids with other critical resistance genes, particularly those encoding ESBLs such as bla_*CTX–M and blaNDM*_ (Kaewnirat et al., 2022; Zhang et al., 2025). FosA3 is most commonly found on conjugative plasmids that carry CTX-M subtype ESBL genes. It may be cotransferred via shared mobile genetic elements (He et al., 2013; Loayza-Villa et al., 2020; Yao et al., 2016), implying that the selective pressure exerted by one class of antibiotics (such as cephalosporins or carbapenems) can coselect for and promote the dissemination of plasmids that also carry fos genes, enabling pathogens to develop resistance to multiple antibiotics simultaneously, thereby severely compromising our therapeutic arsenal. Consequently, fosfomycin combination therapy is not only a sound therapeutic option but also an imperative strategy for combating pathogens harboring multidrug-resistant plasmids.

## 4 Combination therapy with fosfomycin to combat bacterial resistance

### 4.1 Synergy with β-lactams: sequential inhibition of cell wall synthesis

**FIGURE 2:**
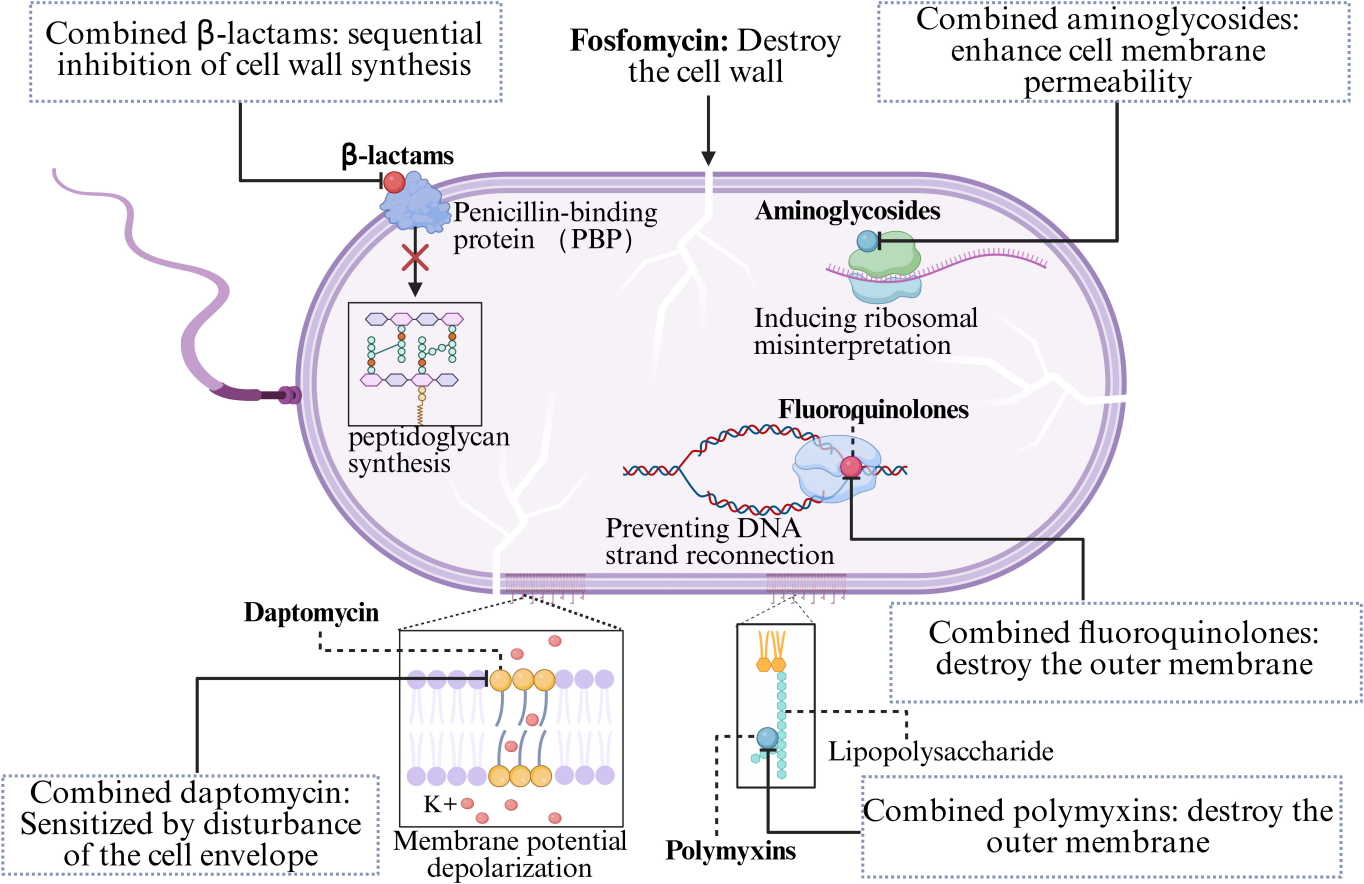
The antibacterial mechanism of fosfomycin in combination with other antibiotics.

β-Lactam antibiotics (carbapenems, cephalosporins, penicillins, and monobactams) constitute an exceptionally broad class of antibiotics characterized by a β-lactam ring in their chemical structure, which inhibits peptidoglycan synthesis (Flores-Kim et al., 2019). The β-lactam ring binds to the active site of penicillin-binding proteins (PBPs), thereby blocking PBP-catalyzed transpeptidation (cross-linking), which results in weakened bacterial cell walls and subsequent cell lysis (Flores-Kim et al., 2019; Mora-Ochomogo and Lohans, 2021). Due to their safety profile and broad spectrum of activity, β-lactams and newer β-lactamase inhibitor combinations remain among the most reliable and effective antibiotic classes globally for treating both simple and severe infections (Venuti et al., 2023). However, the rapid global spread of resistance challenges the efficacy of single antibiotic classes (Munoz-Price et al., 2013). Since fosfomycin inhibits the initial and critical step of bacterial cell wall synthesis (the MurA enzyme), it renders the cell wall biosynthesis system in a “fragile” state, characterized by enhanced permeability. In contrast, β-lactams exert their effect by irreversibly binding to PBPs, thereby inhibiting the final cross-linking/transpeptidation steps. This “front-and-rear attack” strategy induces severe cell wall stress and increases bacterial susceptibility to lysis, resulting in rapid and synergistic bactericidal activity (Figure 2; Bakthavatchalam et al., 2020; Silver, 2017). Furthermore, fosfomycin-induced cell wall stress may modulate bacterial stress response pathways, altering the expression and distribution of cell membrane and cell wall components, which in turn improves the accessibility of β-lactams to their PBPs. Additionally, studies have shown that the combination of fosfomycin and β-lactams leads to a more pronounced reduction in the functional expression or activity of PBPs, further elucidating the mechanistic basis for their synergistic antibacterial effect (del Río et al., 2016).

#### 4.1.1 Combinations with carbapenems

Carbapenems (such as meropenem and imipenem) are considered last-resort antibiotics for treating bacterial infections. They are widely used to manage severe infections, including hospital-acquired pneumonia (HAP), complicated intra-abdominal infections (cIAIs), and bloodstream infections (BSIs) (Zhang S. et al., 2024). Consequently, resistance to carbapenems poses a serious clinical challenge. Substantial evidence has demonstrated that combination therapy with fosfomycin and carbapenem antibiotics has significant synergistic effects and has potential for combating resistant bacteria (Adaleti et al., 2023; Al-Quraini et al., 2022).

Meropenem (MEM), a broad-spectrum carbapenem with potent activity against gram-negative bacteria, has synergistic potential with fosfomycin against carbapenem-resistant Enterobacterales (CRE). For example, Al-Quraini et al. (2022) used a FOS-MEM combination against extensively drug-resistant (XDR) and pandrug-resistant (PDR) *Klebsiella pneumoniae*. Despite harboring a formidable array of resistance genes, including β-lactamases (*bla*_*SHV–11*_, *bla*_*TEM–1b*_, *bla*_*CTX–M–15*_, *bla*_*OXA–232*_) and fosfomycin resistance determinants (*fosA5*, *fosA6*, mutated *uhpT*), excellent combination therapy (94.4%) with a fractional inhibitory concentration index (FICI) ≤ 0.50 was observed. Another study used the same combination against carbapenemase-producing *K. pneumoniae* (KPC-Kp), which reduced the meropenem MIC by 8- to 2048-fold, restoring meropenem susceptibility in 82.4% of the isolates (Ribeiro et al., 2023). Furthermore, the FOS-MEM combined regimen achieved 100% survival in a *Galleria mellonella* larval model. Albiero et al. (2016) also demonstrated a good combination regimen with FOS-MEM against KPC-2-producing *K. pneumoniae*, reducing both meropenem and fosfomycin MICs to susceptible ranges. Additionally, a recent clinical case reported the successful cure of postneurosurgical ventriculitis caused by KPC-producing *K. pneumoniae* via a combination of meropenem/vaborbactam (MVB) and intravenous fosfomycin (Volpicelli et al., 2024), further confirming the clinical utility of FOS-MEM combinations. In addition to *K. pneumoniae*, fosfomycin combined with meropenem also demonstrates effective synergistic bactericidal activity *in vitro* and *in vivo* against other Gram-negative pathogens, including β-lactamase-producing *P. aeruginosa* and carbapenem-resistant *E. coli* (Albiero et al., 2019; Drusano et al., 2018; Suich et al., 2022).

The imipenem (IPM)-fosfomycin combination has similar synergistic potential against carbapenem-resistant gram-negative bacteria. Studies have reported that FOS-IPM achieves synergistic effects against carbapenem-resistant *Acinetobacter baumannii* (CRAB) in 65.2% of cases, indicating that FOS-IPM is more effective than imipenem combined with other antibiotics, even in fosfomycin-resistant isolates (MIC ≥ 64 mg/L) (Singkham-In and Chatsuwan, 2018). Another study revealed that the combination of imipenem-relebactam with fosfomycin had a synergistic effect on 60% of 100 carbapenem-resistant gram-negative isolates and was additive on 40%, exhibiting synergistic activity against all the tested *K. pneumoniae* and *A. baumannii* isolates (Xu et al., 2022). Among the carbapenem-resistant *Acinetobacter calcoaceticus-baumannii* (ACB) complex isolates, while fosfomycin monotherapy resulted in insufficient killing of *A. baumannii*, *A. pittii*, and *A. nosocomialis*, the FOS-IPM combination significantly enhanced antibacterial efficacy, with a FICI of ≤0.5 (Singkham-In and Chatsuwan, 2022), demonstrating its utility against carbapenem-resistant ACB infections. Furthermore, a clinical case highlights FOS-IPM as a potential salvage therapy for MRSA infections (Nakamura et al., 2020). Continuous administration of imipenem/cilastatin (1.5 g daily) and fosfomycin (4.0 g daily) for 4 weeks improved vertebral osteomyelitis and a psoas abscess caused by MRSA, with *E*-test combination therapy tests confirming the optimal co-administration combination of imipenem/cilastatin and fosfomycin. Collectively, these studies demonstrate the potential of fosfomycin as an adjuvant to carbapenems, which jointly inhibit cell wall synthesis and reduce the rates of resistance.

#### 4.1.2 Combinations with cephalosporins and β-lactamase inhibitors

Compared with other types of antibiotics, cephalosporins represent a highly efficacious and indispensable class of β-lactam antibiotics, offering a broader spectrum of antimicrobial activity and fewer side effects, and are often utilized for mild to severe infectious diseases (Lin and Kück, 2022). However, the overuse and misuse of cephalosporins for prophylaxis, therapy, or food production have significantly contributed to the emergence of numerous drug-resistant pathogens. The development of β-lactamase inhibitors has helped preserve the efficacy of β-lactams against β-lactamase-producing pathogens. Novel cephalosporin/β-lactamase inhibitor combinations, such as ceftazidime/avibactam (CZA) and ceftolozane/tazobactam (C/T), are particularly important for treating MDR gram-negative pathogens (Katsarou et al., 2025).

Ceftazidime/avibactam has been employed as a first-line therapy for carbapenem-resistant *K. pneumoniae* (CRKP) infections. However, it is notable that the rate of resistance to CZA is also increasing (Wang et al., 2020; Zhang et al., 2020). Studies have revealed that the combination of CZA with FOS restores antimicrobial susceptibility in MDR *P. aeruginosa* by targeting PBP3, *Pseudomonas*-derived cephalosporinase (PDC), and MurA (Winkler et al., 2015). Subsequently, Papp-Wallace et al. (2019) demonstrated the efficacy of the CZA-FOS combination against the MDR *P. aeruginosa* clinical isolate CL232, which harbored mutations conferring β-lactam resistance and exhibited upregulated expression of *bla*_*PDC*_, the *mexAB-oprM* efflux pump, and *murA*. Combination regimen with CZA-FOS reduced the frequency of resistance to either single agent. Furthermore, Tüzemen et al. (2024) reported that the FOS-CZA combination exhibited enhanced antibacterial activity against CRKP, achieving combination treatment in 63.6% of isolates, with susceptibility rates of 89.1% to CZA and 47.3% to FOS among CRKP isolates. Another study reported similar synergistic results (>60%) when the FOS-CZA combination was used against MBL-producing *K. pneumoniae* (Wu et al., 2024). Unlike ceftazidime/avibactam, ceftolozane/tazobactam (C/T) has enhanced activity against certain AmpC β-lactamases and *P. aeruginosa* (Cho et al., 2015). The synergistic effect of C/T and FOS against MDR *P. aeruginosa* was reported to be 88.9% (24/27), reducing the C/T MIC by 3- to 9-fold, despite the strains being resistant to C/T and FOS individually (Cuba et al., 2020). Attwood et al. (2023) demonstrated that adding fosfomycin or tobramycin to C/T at simulated human clinically observed concentrations reduced the bacterial burden and the risk of resistance emergence in *P. aeruginosa* isolates with MICs at or above the clinical breakpoint (MIC ≥ 4 mg/L).

Although promising *in vitro* data exist for the use of fosfomycin combined with cephalosporins against MDR pathogens, clinical cases reporting on this combination appear limited. In one case, clinical cure was achieved in a patient with post-neurosurgical ventriculitis caused by severe, refractory MDR *P. aeruginosa* (FOS MIC = 64 mg/L) treated with a continuous infusion of CZA and FOS, and reached optimal PK/PD targets (Gatti et al., 2022). However, a retrospective study reported mortality in all six patients with *P. aeruginosa* infections treated with the combination of FOS and CZA (Anastasia et al., 2023). Therefore, MDR *P. aeruginosa*, particularly in immunocompromised patients and despite susceptibility at high doses, remains a significant threat. Further studies are warranted to optimize combination therapy with fosfomycin and confirm its efficacy.

### 4.2 Synergy with aminoglycosides: enhanced cell membrane permeability

Aminoglycoside antibiotics (amikacin, tobramycin, gentamicin) constitute a vital component of the therapeutic arsenal for certain bacterial infections, particularly those caused by aerobic, Gram-negative pathogens, characterized by their broad spectrum, rapid action, and low allergenic potential (Le et al., 2023). The antibacterial mechanism involves the irreversible binding of the compound to the bacterial 30S ribosomal subunit, resulting in misreading during protein translation. This results in the production of aberrant proteins, which further disrupts cell membrane integrity, leading to the leakage of cellular contents and facilitating a massive influx of aminoglycoside molecules into the cytoplasm, ultimately resulting in rapid, concentration-dependent bactericidal killing (Davis, 1988; Taber et al., 1987). When combined with fosfomycin, the inhibition of cell wall synthesis by fosfomycin compromises bacterial cell membrane integrity, thereby increasing bacterial permeability (Figure 2). This facilitates the enhanced entry of aminoglycosides into the cell, allowing greater access to their ribosomal targets.

Reports of *in vitro* combination therapy between fosfomycin and aminoglycosides are highly consistent. Studies have demonstrated significant synergistic effects of fosfomycin combined with amikacin or isepamicin against resistant *P. aeruginosa*, markedly reducing aminoglycoside MICs by up to 64-fold or more (Cai et al., 2009). This not only restores susceptibility in highly resistant strains but also allows for the use of lower, less toxic doses. Time-kill kinetics confirmed the synergistic bactericidal activity of fosfomycin combined with aminoglycosides (amikacin, gentamicin, and tobramycin) against fosfomycin-resistant *A. baumannii* (MIC ≥ 128 μg/mL), resulting in >99.9% bacterial reduction and a 2- to 16-fold decrease in the fosfomycin MIC (Nwabor et al., 2021). Furthermore, the combination of fosfomycin and gentamicin was shown to act synergistically against *E. coli* biofilm strains (Wang et al., 2019). In a hollow-fiber infection model (HFIM), co-administration with fosfomycin (8 g/8 h) and amikacin (15 mg/kg every 24 h) achieved rapid eradication of fosfomycin-heteroresistant *E. coli* cultures, whereas neither fosfomycin nor amikacin monotherapy was effective in sterilizing the cultures (Portillo-Calderón et al., 2023). These results further support the notion that combinations of aminoglycosides and fosfomycin can rapidly reduce the bacterial burden and prevent the emergence of resistant subpopulations to antibiotics such as fosfomycin.

In *in vivo* studies, the combination of fosfomycin and aminoglycosides also demonstrated synergistic antibacterial activity. In a murine peritoneal sepsis model, the combination of fosfomycin and amikacin reduced the spleen concentrations of VIM-1-producing and OXA-48 plus CTX-M-15-producing *K. pneumoniae*, whereas the combination of fosfomycin and gentamicin reduced the spleen concentrations of KPC-3-producing strains (Cebrero-Cangueiro et al., 2021). Notably, none of these combinations improved survival in mice infected with carbapenemase-producing *K. pneumoniae* strains. Therefore, the evaluation of combinations of fosfomycin and aminoglycosides for other types of infections warrants further consideration. In patients with cystic fibrosis and *Pseudomonas* airway infection, fosfomycin/tobramycin inhalation (FTI) significantly improved the predicted FEV1% observed during an aztreonam inhalation solution (AZLI) run-in period and was well tolerated (Trapnell et al., 2012). These findings demonstrate that FTI is a promising therapeutic option for anti-*Pseudomonas* treatment in cystic fibrosis patients. Additionally, a novel composite susceptibility breakpoint threshold for predicting successful combination therapy has been proposed: when the product of the MICs of two drugs is less than 256, successful co-administration can be predicted (Darlow et al., 2021), representing a significant conceptual advancement beyond simple FICI values, offering a more clinically translatable, PK/PD-based threshold for predicting the efficacy of combination therapies.

### 4.3 Synergy with fluoroquinolones: disrupting the outer membrane

Fluoroquinolone antibiotics (ciprofloxacin, levofloxacin) are potent, synthetically derived broad-spectrum antibacterial agents that exhibit bactericidal activity against both gram-positive and gram-negative bacteria (Rusu et al., 2023). The mechanism relies on targeting two enzymes essential for DNA replication and repair: DNA gyrase and topoisomerase IV (Kabbani et al., 2018; Werner et al., 2011). Fluoroquinolones block DNA strand rejoining by reversibly and noncovalently binding to the cleavage complex at the cleavage-ligation active site, thereby compromising DNA replication (Correia et al., 2017). These irreparable DNA strand breaks trigger the bacterial SOS response, followed by a protein cascade that ultimately leads to bacterial death (Blondeau, 2004). The synergistic mechanism of combining fluoroquinolones with FOS involves the disruption of the bacterial outer membrane and cell wall integrity by FOS (Figure 2), enhancing the penetration of fluoroquinolones and increasing their bactericidal efficiency.

However, studies have reported that the fluoroquinolone ciprofloxacin (CIP) can disrupt the outer membrane structure itself. Combined CIP and FOS treatment induced significant morphological changes, and adding CIP first rather than FOS first produced a stronger synergistic effect on CIP-resistant *P. aeruginosa* (Yamada et al., 2007). In a study of CIP-resistant *Shigella* isolates, the CIP-FOS combination demonstrated synergistic effects both *in vitro* and in a *Galleria mellonella* larval model, significantly improving larval survival (Liu et al., 2019). Furthermore, studies indicate that combined FOS-CIP therapy reduces the incidence of postoperative infectious complications. Following transrectal ultrasound-guided prostate biopsy (TRUSPB), co-administration of CIP and FOS was associated with fewer infectious complications (0.3%), suggesting its potential applicability in the Era of high rectal flora resistance (Lim et al., 2021). A retrospective study revealed that the primary infectious outcome after prostate needle biopsy (PNB) was urosepsis. The incidence of urosepsis was 1.1% (12/1090) with CIP alone, which was reduced to 0.2% (2/1197) with CIP-FOS combination therapy (Morin et al., 2020). Additionally, Gómez-Garcés et al. (2017) described a case of infection involving surgical mesh by a carbapenemase-producing *Enterobacter cloacae* strain that was resistant to various antimicrobial regimens and was successfully treated with a combination of ciprofloxacin and fosfomycin. Collectively, these results demonstrate the prophylactic and synergistic antibacterial potential of ciprofloxacin-fosfomycin combination therapy against resistant strains, particularly CIP-resistant Enterobacterales.

### 4.4 Synergy with polymyxins: disrupting the outer membrane

Polymyxins are polypeptide antibiotics that target aerobic, Gram-negative pathogens; however, only two are currently available: polymyxin E (colistin) and polymyxin B (El-Sayed Ahmed et al., 2020). The core mechanism involves the binding of the hydrophilic (positively charged) moiety of polymyxin molecules to the anionic lipopolysaccharide (LPS) in the outer membrane of Gram-negative bacteria, which disrupts the outer membrane’s structure and function, facilitating further penetration into the inner membrane and ultimately leading to bacterial cell death (Kaye et al., 2016). This action significantly enhances FOS uptake into bacterial cells (Figure 2).

Mechanism-based models suggest that, compared to monotherapy, the combination of fosfomycin and polymyxin B (PMB) exhibits greater bactericidal efficacy against KPC-2-producing *K. pneumoniae* (KPC-Kp) (Sharma et al., 2022). Furthermore, the combination therapy achieved greater reductions in cytokine expression. In a hollow-fiber infection model, monotherapy resulted in >3 log_10_ CFU/mL killing of KPC-Kp within 3 h; however, resistant subpopulations regrew and proliferated by 48 h. In contrast, the PMB-FOS combination achieved rapid bactericidal killing (>6 log_10_ CFU/mL reduction) while preventing the emergence of resistance to both PMB and FOS (Bulman et al., 2018). In *P. aeruginosa*, the combination of FOS and PMB increased bacterial killing but did not suppress the emergence of fosfomycin resistance (Walsh et al., 2016). Additionally, Scudeller et al. (2021) described high and moderate synergy rates (high synergy, ES ≥ 0.75; moderate synergy, 0.35 < ES < 0.75) for FOS combined with colistin (COL) against CRKP through a systematic review and meta-analysis. Compared with monotherapy, time-kill assays demonstrated that FOS combined with COL exhibited superior bactericidal activity against KPC-Kp (Zhang J. et al., 2024). The FOS-COL combination also increased the survival of KPC-Kp-infected Thp-1 cells while reducing their cytotoxicity and resistance rates. Moreover, this study revealed that the synergistic bactericidal effect of FOS-COL involves the modulation of ROS accumulation and the suppression of ribosomal protein transcription (Zhang J. et al., 2024). Recently, Khalifa et al. (2025) confirmed the synergistic activity of the FOS-COL combination against *E. coli* and *Salmonella* strains via time-kill assays, further supporting its potential against multidrug-resistant *E. coli* and *Salmonella*.

In murine models, combinations of fosfomycin and polymyxin have also demonstrated significant synergistic effects. In an NDM-1-producing *E. coli* mouse peritonitis infection model, compared with monotherapy, the combination of FOS with COL resulted in reduced mortality and lower bacterial counts in the spleen in all strains tested (Le Menestrel et al., 2021). Furthermore, the combination prevented the selection of resistant mutants. In a murine model of multidrug-resistant *A. baumannii* pneumonia, compared with monotherapy, FOS combined with COL significantly reduced lung bacterial loads at 24 and 48 h (Ku et al., 2019). A clinical study further demonstrated that compared with COL monotherapy, FOS combined with COL yielded more favorable microbiological outcomes in patients infected with carbapenem-resistant *A. baumannii* (CRAB), along with trends toward improved clinical outcomes and lower mortality (Sirijatuphat and Thamlikitkul, 2014). Although fosfomycin-polymyxin combinations demonstrate objective efficacy, future multicenter studies with larger patient cohorts are warranted to definitively establish their benefit for patients infected with carbapenem-resistant strains.

### 4.5 Synergy with daptomycin: resensitization via cell envelope perturbation

Daptomycin (DAP) is a critical cyclic lipopeptide antibiotic used to treat infections caused by gram-positive bacteria (Miller et al., 2016). DAP has a distinct mechanism of action: it binds in a calcium-dependent manner to the bacterial membrane lipids phosphatidylglycerol (PG) and cardiolipin (CL), inserts into the lipid bilayer, and forms transmembrane ion channels, resulting in rapid depolarization of the membrane potential and subsequent bactericidal killing (Huang, 2020). In recent years, reports of daptomycin resistance in some gram-positive pathogens (such as *S. aureus*, *Enterococcus faecium*, and *Enterococcus faecalis*) have increased. In daptomycin-resistant MRSA and VRE, resistance is often associated with adaptive changes in the cell envelope, such as increased surface positive charge, altered membrane fluidity, and redistribution of anionic cardiolipin microdomains. Fosfomycin appears to counteract these adaptations by disrupting the bacterial membrane potential (Hall Snyder et al., 2016; Mishra et al., 2022). It perturbs the cell envelope, reducing the net surface positive charge and altering membrane fluidity and cardiolipin localization, thereby resensitizing bacteria to the membrane-depolarizing effects of daptomycin (Figure 2).

Numerous *in vitro* studies have reported significant synergy between FOS and DAP against MRSA, with synergy rates of up to 100% (Aktas and Derbentli, 2017; Saravolatz and Pawlak, 2022). This combination not only enhances the killing of both daptomycin-susceptible and daptomycin-resistant MRSA but also effectively prevents the evolution of resistance and resensitizes resistant strains (Mishra et al., 2022). Recently, Ai et al. (2025) reported that the FOS-DAP combination therapy against MRSA suppressed the emergence of resistance-conferring mutations and lowered the minimum inhibitory concentration of each drug in mutants. They noted that mutations in the *mprF* and *murA* genes were detected in the DAP and FOS monotherapy groups, respectively, whereas no such mutations were found in the combination group. These findings provide crucial insights into preventing the evolution of MRSA resistance.

Furthermore, DAP combined with FOS demonstrated superior antibacterial and antibiofilm effects against both planktonic and adherent linezolid-resistant *E. faecalis* isolates (Zheng et al., 2019). DAP is approved for treating MRSA bacteremia and endocarditis in the clinic (Shaw et al., 2015). A randomized clinical trial demonstrated a 12% higher treatment success rate for patients with MRSA bacteremia who received DAP-FOS compared to those who received DAP alone, with fewer cases of clinical or microbiological failure observed in the combination group (Pujol et al., 2021). Another clinical trial reported that FOS-DAP co-administration reduced mortality by 17.8% in patients with vancomycin-resistant *Enterococcus* (VRE) bloodstream infections, with particularly significant effects in patients with lower Pitt bacteremia scores or FOS MICs ≤ 64 mg/L (Tseng et al., 2023). The combination of DAP and FOS was also shown to be synergistic and rapidly bactericidal against MRSA in a rabbit model of experimental endocarditis (García-de-la-Mària et al., 2018).

However, combined regimens are often accompanied by adverse events, which can reduce treatment success and limit clinical utility. Hypernatremia and hypokalemia, which are related to sodium overload, are the most common concerns. Studies indicate that compared with daptomycin monotherapy, fosfomycin combination therapy leads to higher rates of hypernatremia and hypokalemia (Tseng et al., 2023). The high sodium salt content of fosfomycin is likely responsible for the significantly increased incidence of hypernatraemia. Hypokalemia appears to be associated with increased renal excretion of fosfomycin in the distal renal tubules. Consequently, optimizing the pharmacodynamics of fosfomycin combination regimens is imperative for future use. Table 2 summarizes the key studies on fosfomycin combination therapy.

**TABLE 2:** Summary of key studies on combination therapy with fosfomycin.

Combination antibiotics		Primary target pathogen(s)	Presumed synergistic mechanism	Synergy results	References
Carbapenems	Meropenem	XDR and PDR *K. pneumoniae*	Sequential inhibition of cell wall synthesis	Synergistic effect of 94.4%, FICI ≤ 0.5, improving the sensitivity of drug-resistant bacteria to antibiotics.	Al-Quraini et al., 2022
Carbapenems	Meropenem	Carbapenemase-producing *K. pneumoniae*	Sequential inhibition of cell wall synthesis	Reduced the MIC of meropenem by 8 to 2048 times, restoring the activity of 82.4% of the isolated strains against meropenem.	Ribeiro et al., 2023
Carbapenems	Meropenem	Carbapenemase-producing *K. pneumoniae*	Sequential inhibition of cell wall synthesis	A synergistic effect was achieved, with an FICI of 0.3, thereby reducing the overall antibiotic MIC to the sensitive range.	Della Rocca et al., 2023
Carbapenems	Meropenem	β-lactamase-producing *P. aeruginosa*	Sequential inhibition of cell wall synthesis	Antibiotic MIC reduced by 8 times; 68% of isolates achieved PTA.	Albiero et al., 2019
Carbapenems	Meropenem	Multidrug-resistant *P. aeruginosa*	Sequential inhibition of cell wall synthesis	Promotes bacterial killing of >6 log_10_ CFU/ml, reversing resistance to fosfomycin and meropenem.	Drusano et al., 2018
Carbapenems	Meropenem/ vabotapan	Carbapenemase-producing *K. pneumoniae*	Sequential inhibition of cell wall synthesis	Cure infectious thrombosis caused by KPC-Kp.	Oliva et al., 2021
Carbapenems	Meropenem	Multidrug-resistant *Salmonella*	Sequential inhibition of cell wall synthesis	Fosfomycin, combined with high-dose meropenem, successfully treats *Salmonella enterica* serotype typhimurium infections.	Kleine et al., 2017
Carbapenems	Imipenem	Carbapenem-resistant *A. baumannii*	Sequential inhibition of cell wall synthesis	Synergistic effect of 65.2%.	Singkham-In and Chatsuwan, 2018
Carbapenems	Imipenem	*A. baumannii*, *A. pittii*, and *A. nosocomialis*	Sequential inhibition of cell wall synthesis	It exhibits an antibacterial synergistic effect, with an FICI of ≤0.5.	Singkham-In and Chatsuwan, 2022
Carbapenems	Imipenem	Carbapenemase-producing *K. pneumoniae*	Sequential inhibition of cell wall synthesis	It shows an additive effect on KPC-Kp.	Yu et al., 2017
Carbapenems	Imipenem	Multidrug-resistant *E. coli*	Sequential inhibition of cell wall synthesis	7/8 drug-resistant bacteria are sensitive to the combination of FOS-IMP.	El-Wafa and Ibrahim, 2020
Carbapenems	Imipenem	MRSA and glycopeptide-intermediate resistant *S. aureus*	Sequential inhibition of cell wall synthesis	The FOF + IPM group showed the best antibacterial activity, significantly reducing PBP1 and PBP2.	del Río et al., 2016
Carbapenems	Imipenem/ cilastatin	MRSA	Sequential inhibition of cell wall synthesis	Improvement of vertebral osteomyelitis and psoas abscess caused by MRSA.	Nakamura et al., 2020
Cephalosporins and β-lactamase inhibitors	Ceftazidime/ avibactam	Multidrug-resistant *P. aeruginosa*	Sequential inhibition of cell wall synthesis	Combination therapy reduced the frequency of resistance to either single agent.	Papp-Wallace et al., 2019
Cephalosporins and β-lactamase inhibitors	Ceftazidime/ avibactam	β-lactamase-producing *K. pneumoniae*	Sequential inhibition of cell wall synthesis	The synergistic effect of strains producing β-lactamase is more than 60%, reducing the MIC of FOS and CZA.	Wu et al., 2024
Cephalosporins and β-lactamase inhibitors	Ceftazidime/ avibactam	Multidrug-resistant *E. coli*	Sequential inhibition of cell wall synthesis	Inhibits the growth of drug-resistant bacteria and reduces the MIC of CAZ (6-fold) and FOS (16-fold).	Kroemer et al., 2024
Cephalosporins and β-lactamase inhibitors	Ceftazidime/ avibactam	Multidrug-resistant *P. aeruginosa*	Sequential inhibition of cell wall synthesis	Successful cure of a patient with neurosurgical posterior ventriculitis caused by severe, refractory, drug-resistant *P. aeruginosa*.	Gatti et al., 2022
Cephalosporins and β-lactamase inhibitors	Ceftolozane/ tazobactam	Multidrug-resistant *P. aeruginosa*	Sequential inhibition of cell wall synthesis	Synergistic effect of 88.9%, reducing the C/T MIC by 3- to 9-fold.	Cuba et al., 2020
Cephalosporins and β-lactamase inhibitors	Ceftolozane/ tazobactam	*P. aeruginosa*	Sequential inhibition of cell wall synthesis	Reduce the bacterial burden and the risk of resistance emergence in *P. aeruginosa* isolates with MICs at or above the clinical breakpoint (MIC ≥ 4 mg/L).	Attwood et al., 2023
Aminoglycosides	Amikacin	*P. aeruginosa*	Enhanced cell membrane permeability	Reduces MIC by 64 times or more, restoring sensitivity of resistant strains.	Cai et al., 2009
Aminoglycosides	Amikacin, gentamicin, tobramycin	Multidrug-resistant *A. baumannii*	Enhanced cell membrane permeability	Reduce the MIC of fosfomycin by 2-to 16-fold, resulting in a reduction of bacteria by more than 99.9%.	Nwabor et al., 2021
Aminoglycosides	Gentamicin	*E. coli*	Enhanced cell membrane permeability	Collaborative anti-biofilm.	Wang et al., 2019
Aminoglycosides	Gentamicin	Carbapenemase-producing *K. pneumoniae*	Enhanced cell membrane permeability	Reduce the concentration of KPC-3-producing *K. pneumoniae* in the spleen.	Cebrero-Cangueiro et al., 2021
Aminoglycosides	Amikacin	Drug-resistant *E. coli*	Enhanced cell membrane permeability	Combination therapy demonstrates the rapid eradication of fosfomycin-heteroresistant *E. coli* cultures.	Portillo-Calderón et al., 2023
Aminoglycosides	Amikacin	Carbapenemase-producing *E. coli*	Enhanced cell membrane permeability	Create a cumulative effect.	Yu et al., 2022
Aminoglycosides	Amikacin	Colistin-resistant *K. pneumoniae*	Enhanced cell membrane permeability	Fosfomycin (8 g/8 h) and amikacin (15 mg/kg once daily) can effectively kill drug-resistant bacteria to the greatest extent.	Yu et al., 2018
Aminoglycosides	Amikacin	Carbapenemase-producing *K. pneumoniae*	Enhanced cell membrane permeability	Reduce the concentration of VIM-1 and OXA-48 plus CTX-M-15-producing *K. pneumoniae* in the spleen.	Cebrero-Cangueiro et al., 2021
Aminoglycosides	Tobramycin	*Pseudomonas*	Enhanced cell membrane permeability	Fosfomycin/tobramycin inhalation maintained the significant improvement in predicted FEV (1) % observed during the run-in period with aztreonam inhalation solution and was well tolerated.	Trapnell et al., 2012
Fluoroquinolones	Ciprofloxacin	CIP-resistant *P. aeruginosa*	Disrupting the outer membrane	CIP and FOS together cause significant morphological changes.	Yamada et al., 2007
Fluoroquinolones	Ciprofloxacin	CIP-resistant *Shigella*	Disrupting the outer membrane	Showed synergistic effects *in vitro* and the *Galleria mellonella* larval model, significantly improving larval survival rates.	Liu et al., 2019
Fluoroquinolones	Ciprofloxacin	Drug-resistant *E. coli*	Disrupting the outer membrane	Combination therapy reduces infection complications after prostate biopsy.	Lim et al., 2021; Morin et al., 2020
Fluoroquinolones	Ciprofloxacin	Carbapenemase-producing *Enterobacter cloacae*	Disrupting the outer membrane	Successful treatment of a patient with surgical mesh infection	Gómez-Garcés et al., 2017
Polymyxins	Polymyxin B	Carbapenemase-producing *K. pneumoniae*	Disrupting the outer membrane	Produces stronger bactericidal effects against KPC-Kp and reduces cytokine expression.	Sharma et al., 2022
Polymyxins	Polymyxin B	Carbapenemase-producing *K. pneumoniae*	Disrupting the outer membrane	Demonstrates rapid bactericidal activity, preventing the spread of polymyxin B and fosfomycin resistance.	Bulman et al., 2018
Polymyxins	Polymyxin B	*P. aeruginosa*	Disrupting the outer membrane	Combination therapy can enhance bacterial killing, but it cannot prevent the emergence of fosfomycin resistance.	Walsh et al., 2016
Polymyxins	Colistin	Carbapenemase-producing *K. pneumoniae*	Disrupting the outer membrane	High and moderate synergistic efficacy against carbapenem-resistant *K. pneumoniae*.	Scudeller et al., 2021
Polymyxins	Colistin	Carbapenemase-producing *K. pneumoniae*	Disrupting the outer membrane	Exerts synergistic bactericidal effects by regulating ROS accumulation and inhibiting ribosomal protein transcription.	Zhang J. et al., 2024
Polymyxins	Colistin	Carbapenemase-producing *E. coli*	Disrupting the outer membrane	Reduce mortality in mice infected with peritonitis and lower bacterial load.	Le Menestrel et al., 2021
Polymyxins	Colistin	Multidrug-resistant *A. baumannii*	Disrupting the outer membrane	Reduce bacterial load in the lungs 24 h and 48 h after infection.	Ku et al., 2019
Polymyxins	Colistin	Carbapenem-resistant *A. baumannii*	Disrupting the outer membrane	Reducing mortality in patients infected with carbapenem-resistant *A. baumannii*.	Sirijatuphat and Thamlikitkul, 2014
Daptomycin	\	MRSA	Resensitization via cell envelope perturbation	Synergy effect reaches 100%.	Aktas and Derbentli, 2017; Saravolatz and Pawlak, 2022
Daptomycin	\	MRSA	Resensitization via cell envelope perturbation	Inhibit drug resistance gene mutations and reduce the mutation prevention concentration of each drug.	Ai et al., 2025
Daptomycin	\	Linezolid-resistant *E. faecalis*	Resensitization via cell envelope perturbation	Synergistic antibacterial and anti-biofilm.	Zheng et al., 2019
Daptomycin	\	MRSA	Resensitization via cell envelope perturbation	Combination therapy improves the success rate in patients with bacteremia by 12% compared to daptomycin.	Pujol et al., 2021
Daptomycin	\	Vancomycin-resistant *Enterococcus*	Resensitization via cell envelope perturbation	Reduced mortality rate by 17.8% in patients with VRE bloodstream infections	Tseng et al., 2023
Daptomycin	\	MRSA	Resensitization via cell envelope perturbation	Rapid eradication of MRSA in a rabbit model of experimental endocarditis	García-de-la-Mària et al., 2018

## 5 Clinical application and translational challenges

Despite promising preclinical data for fosfomycin combination therapy, significant obstacles impede the effective translation of these findings into routine clinical practice. This section critically examines these challenges.

### 5.1 Clinical evidence from case reports and trials

Although reports on fosfomycin combination therapies are increasingly encountered in clinical practice, much of the current evidence still originates from isolated case reports or small series. Examples include successful treatment of neurosurgical ventriculitis caused by KPC-producing *K. pneumoniae*, vertebral osteomyelitis due to MRSA, and postoperative infections caused by *Enterobacter cloacae* (Gómez-Garcés et al., 2017; Nakamura et al., 2020; Volpicelli et al., 2024). While these reports are encouraging, they generally lack direct comparison with non-fosfomycin-containing regimens. This limitation restricts the strength of clinical inferences and makes it difficult to attribute outcomes solely to fosfomycin use. Only a limited number of comparative studies are available. For instance, in the context of prostate biopsy prophylaxis, the combination of ciprofloxacin with fosfomycin significantly reduced the incidence of sepsis compared to ciprofloxacin monotherapy (Lim et al., 2021). Similarly, fosfomycin combined with colistin demonstrated a trend toward improved microbiological clearance and reduced mortality in infections caused by carbapenem-resistant organisms (Sirijatuphat and Thamlikitkul, 2014). Furthermore, a randomized clinical trial demonstrated that combining daptomycin with fosfomycin for MRSA bacteremia resulted in a 12% higher success rate compared to daptomycin alone, and led to a 17.8% reduction in mortality in vancomycin-resistant *Enterococcus* bloodstream infections (Pujol et al., 2021). These data provide useful preliminary comparative evidence, but their scope remains limited.

In summary, the clinical evidence supporting fosfomycin combination regimens remains incomplete and often lacks robustness. Due to the observational nature of most studies, heterogeneity in patient populations, and the absence of standardized comparators, the majority of available reports do not permit definitive conclusions. Therefore, it is essential to clearly acknowledge these limitations and conduct larger, multicenter randomized controlled trials to validate the efficacy and safety of fosfomycin-based combinations across various infection types and resistant pathogens.

### 5.2 Efficacy disconnect

A disconnect exists between the *in vitro* and *in vivo* efficacy of fosfomycin combinations, as notably exemplified in cases of *S. aureus* bacteremia. Although *in vitro* studies have demonstrated robust synergy and a solid mechanistic basis for combining fosfomycin with antistaphylococcal agents (such as β-lactams and daptomycin) (Aktas and Derbentli, 2017; del Río et al., 2016; Saravolatz and Pawlak, 2022), a recent systematic review and meta-analysis of three randomized controlled trials (RCTs) by Mourad et al. (2025) concluded that combination therapy did not significantly improve patient mortality (RR 0.85) or reduce rates of persistent bacteremia. This discrepancy may stem from several factors: (1) Patient and infection complexity: Randomized controlled trial (RCT) populations exhibit high heterogeneity, and the complexity of infections (such as endocarditis and deep-seated abscesses) far exceeds that of standardized laboratory models; (2) Suboptimal dosing regimens: The dosing regimens used in trials may fail to achieve the PK/PD targets required for synergy at the infection site; (3) Adverse events (AEs): The meta-analysis revealed a nonsignificant trend toward greater treatment discontinuation due to AEs in the combination arms, which may have potentially confounded the efficacy assessment. In contrast, fosfomycin combinations have a more positive outlook for MDR gram-negative infections. A 2024 review of clinical studies in severe gram-negative infections reported clinical success rates of approximately 75%–80% for fosfomycin combination therapy regimens, even in patients with MDR pathogens (Butler et al., 2024). These findings suggest that fosfomycin combinations can yield synergistic effects that are comparable to or better than those of other common combination therapies.

### 5.3 Standardization gap

The lack of standardization presents a pervasive challenge. First, standardized, readily available susceptibility testing methods are lacking. Both the Clinical and Laboratory Standards Institute (CLSI) and the European Committee on Antimicrobial Susceptibility Testing (EUCAST) recommend agar dilution supplemented with G6P as the reference method for fosfomycin susceptibility testing (Pipitone et al., 2023). However, this method is laborious and time-consuming and is not routinely performed in most clinical laboratories. Alternative methods, such as broth microdilution, *E*-test, and disk diffusion, exhibit poor consistency and lack universal endorsement by authorities, including the CLSI and EUCAST (Smith et al., 2020). Second, validated clinical susceptibility breakpoints are lacking. Clinically validated fosfomycin breakpoints have not been established for many significant pathogens, including *P. aeruginosa* and *A. baumannii* (Zheng et al., 2022). Clinicians and researchers are often compelled to utilize *E. coli* breakpoints and extrapolate to other pathogens, which can be risky. Finally, the impossibility of uniformly defining patient inclusion criteria and microbiological outcomes across different study centers complicates the execution of high-quality, large-scale RCTs. This results in a scarcity of robust RCT data, hindering the establishment of optimal, approved dosing regimens for severe systemic infections. In the absence of optimized dosing and clear evidence of clinical benefit, clinicians remain cautious about the clinical use of these combination regimens.

### 5.4 Dosing dilemma

The dosing of fosfomycin presents a persistent clinical conundrum, largely due to the absence of standardized dosing guidelines. Oral fosfomycin is approved only as a single 3 g dose for uncomplicated urinary tract infections (UTIs), as its bioavailability is insufficient for systemic infections. Systemic infections require high-dose intravenous (IV) fosfomycin (Dijkmans et al., 2017). For severe infections caused by MDR organisms, there is currently no consensus on the optimal IV dosing regimen, with proposals ranging from 8 to 12 g/day for gram-positive pathogens to 16–24 g/day for gram-negative pathogens (Candel et al., 2019). High-dose fosfomycin carries the risk of saline overload (1 g of fosfomycin disodium provides 0.33 g of sodium), potentially causing patient intolerance and treatment discontinuation. Furthermore, in critically ill patients, particularly those with renal impairment, fosfomycin pharmacokinetics exhibit high interindividual variability. This makes standardized dosing unreliable and necessitates PK/PD-guided therapeutic drug monitoring (TDM) to inform individualized dosing (Wu et al., 2024).

## 6 Prospects

As an “old drug,” fosfomycin has gained renewed importance in the current antimicrobial resistance crisis because of its unique mechanism of inhibiting cell wall synthesis and its activity against diverse multidrug-resistant strains. However, its inherent susceptibility to resistance development during monotherapy indicates that its future is inextricably linked to combination regimens. Extensive preclinical research, encompassing *in vitro* studies and animal models, provides compelling evidence in support of various fosfomycin combination regimens. Combinations of β-lactams, aminoglycosides, fluoroquinolones, polymyxins, and daptomycin, which are based on well-defined synergistic mechanisms (such as sequential blockade, enhanced penetration, and resensitization), have demonstrated potent synergistic bactericidal activity and an effective capacity to suppress the development of resistance. These findings offer a solid theoretical foundation and promise for clinical application. Nevertheless, the path from the laboratory to the clinic is fraught with challenges, necessitating further planning for future directions.

**FIGURE 3:**
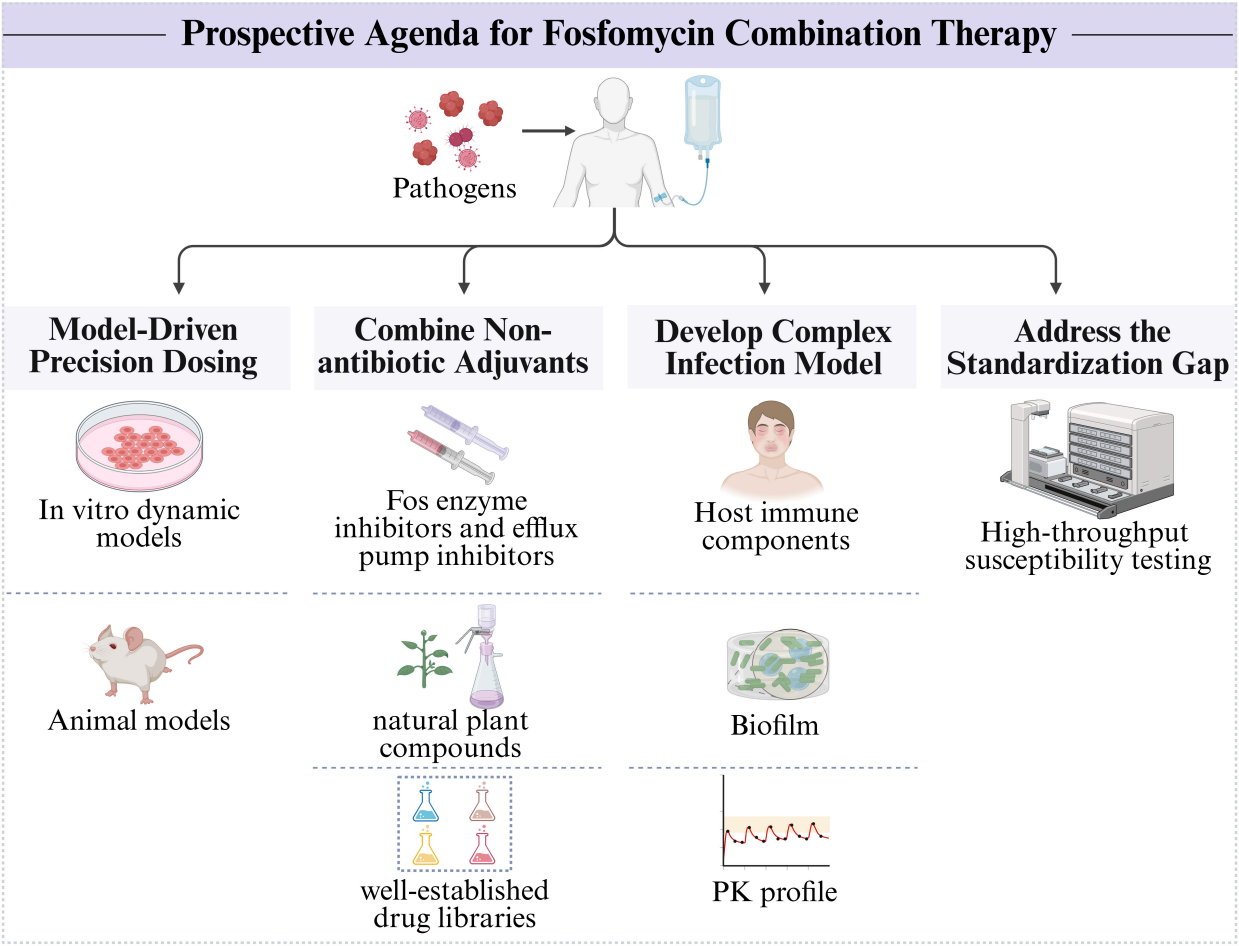
Prospective research agenda for fosfomycin combination therapy.

To unlock the full potential of fosfomycin combination therapy against MDR pathogens and advance its clinical application, we propose a prospective research agenda (Figure 3). First, the future of fosfomycin combination therapy hinges on a transition from empirical dosing to model-informed precision dosing. Future efforts should utilize *in vitro* dynamic models (such as the hollow-fiber infection model) and animal models to comprehensively understand the PK/PD properties of drugs, thereby enabling dosing regimens that provide maximal synergy while minimizing toxicity and resistance development (Wale et al., 2024). Second, the discovery and identification of nonantibiotic adjuvants that potentiate fosfomycin activity (such as FOS enzyme inhibitors and efflux pump inhibitors) to assist in fosfomycin therapy are crucial (Dhanda et al., 2023). High-throughput screening of well-established drug libraries and exploration of natural plant compounds represent important avenues for discovering unexpected synergistic partners (Tiwana et al., 2024). Furthermore, there is a need for improved *in vitro* and *in vivo* models that better simulate the complexity of human infections (such as by integrating host immune components, biofilm formation, and realistic PK profiles) to increase the predictive value of preclinical synergy studies (Wale et al., 2024). Finally, the standardization gap needs to be addressed. Basic researchers can contribute by developing and validating new, more reliable, high-throughput susceptibility testing methods that could form the basis for future clinical standards.
